# Supplementary material for: The interactome of intact mitochondria by cross-linking mass spectrometry provides evidence for coexisting respiratory supercomplexes
Source: Mol Cell Proteomics. 2017 Dec 8;17(2):216–32. doi: 10.1074/mcp.RA117.000470 (PMC5795388; doi:10.1074/mcp.RA117.000470)
Supplement: Supplemental Data [file supp_RA117.000470_133922_0_supp_23978_fzffwf.zip › spectra_annotation/mito_DR_spectra_annotation/149-1-1-1-5-1.pdf]

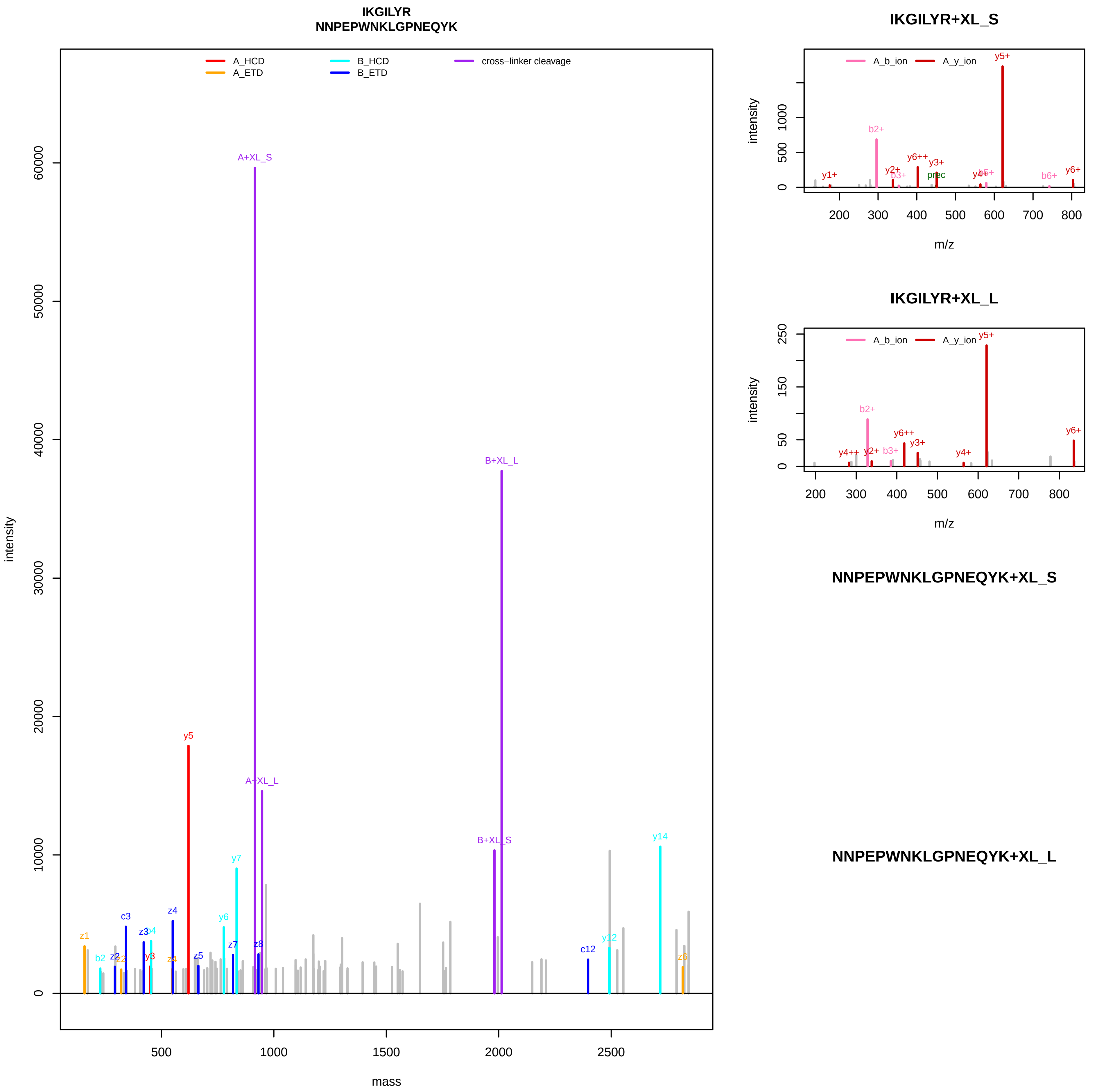
IKGILYR
NNPEPWNKLGPNEQYK
A_HCD
A_ETD
B_HCD
B_ETD
cross−linker cleavage
intensity
mass
0
10000
20000
30000
40000
50000
60000
500
1000
1500
2000
2500
A+XL_S
B+XL_L
A+XL_L
B+XL_S
y5
y14
y7
y12
c12
z1
b2
z2
c3
z3
b4
y3
z4
z5
y6
z7
z8
z6
IKGILYR+XL_S
A_b_ion
A_y_ion
y5+
b2+
y6++
y3+
prec
y2+
b3+
y1+
y4+
b5+
b6+
y6+
m/z
IKGILYR+XL_L
y4++
NNPEPWNKLGPNEQYK+XL_S
NNPEPWNKLGPNEQYK+XL_L
